# Actinomycosis complicating sigmoid diverticular disease: a case report

**DOI:** 10.1186/1757-1626-2-6456

**Published:** 2009-03-10

**Authors:** Aleksandar Vodovnik, Kartik Logishetty

**Affiliations:** 1 Department of Cellular Pathology, Medway Maritime Hospital, ME7 5NY, Gillingham, United Kingdom

## Abstract

A 63-year-old Caucasian woman was admitted to hospital as hypotensive with abdominal tenderness and vaginal discharge. Laboratory investigations showed microcytic anaemia, low albumin and high white cell count. Computerised tomography scans revealed small bowel dilatation, sigmoid diverticula, ascites and pelvic fluid. The endometrial pipelle was positive and vaginal swab was negative for actinomyces. Post mortem examination revealed widespread sigmoid diverticular disease and bowel perforation with an intense inflammation. Actinomycotic granules were noted in the diverticular inflammatory debris, pelvic abscess and lung sections. Clinical course and histomorphological findings favour the perforating sigmoid diverticular actinomycosis as an origin of the systemic infection.

## Introduction

Abdominal and pelvic infections account for 10-20% of reported cases of actinomycosis. The disease presents typically as a slowly growing mass in the ileo-cecal region and patients usually have a history of bowel surgery or ingestion of foreign bodies [1-3]. We present a case of actinomycosis complicating sigmoid diverticular disease.

## Case presentation

**Figure 1:**
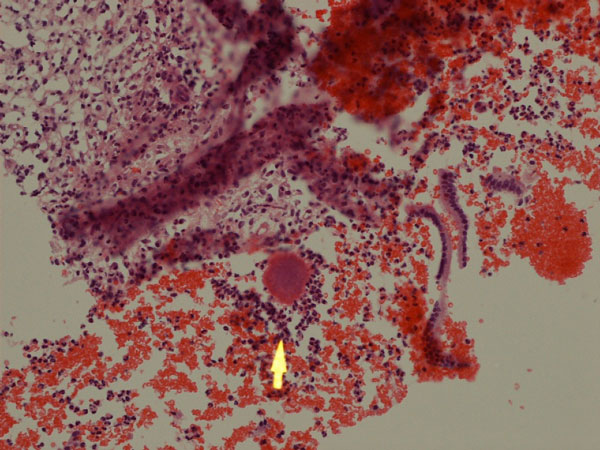
**Endometrial sample showing active inflammation and actinomycotic granules (arrow)**. Hematoxylin and Eosin, 100X.

**Figure 2:**
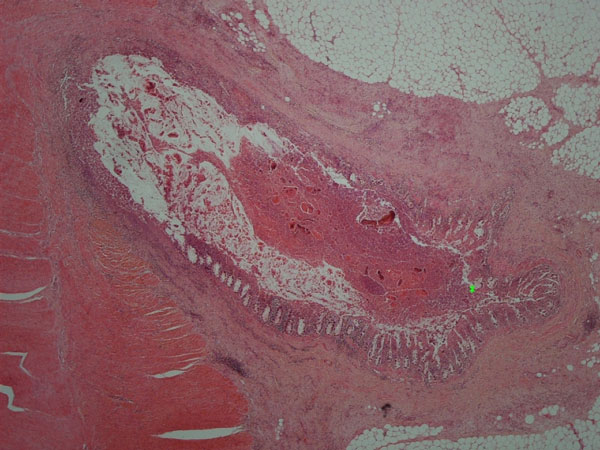
**Sigmoid diverticulitis (star indicates zoom-up site for Figure **3). Hematoxylin and Eosin, 20X.

**Figure 3:**
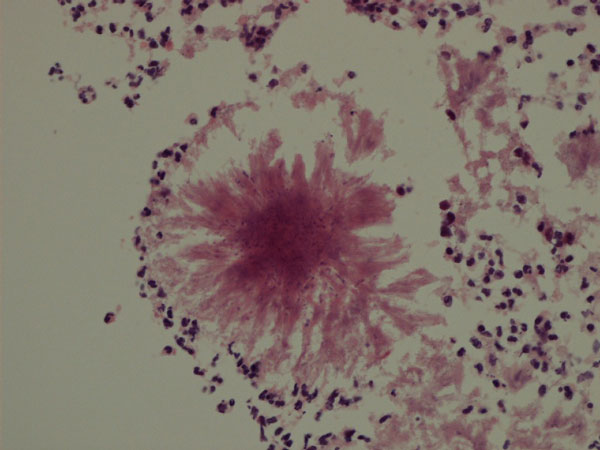
**Diverticular inflammatory debris containing actinomycotic granules**. Hematoxylin and Eosin, 200X.

**Figure 4:**
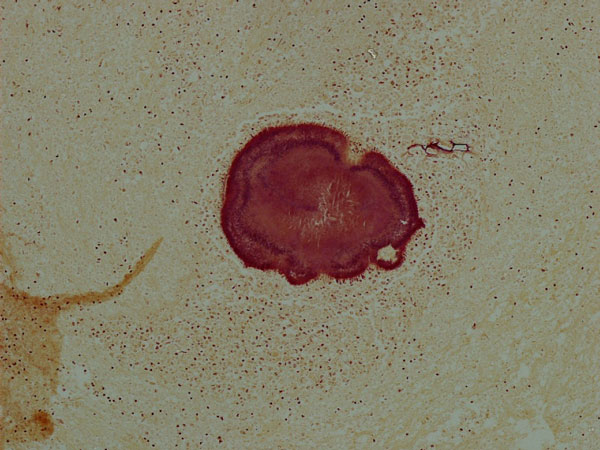
**Actinomycotic granules and food remnants from pelvic abscess**. Brown-Hopps stain, 100X.

**Figure 5:**
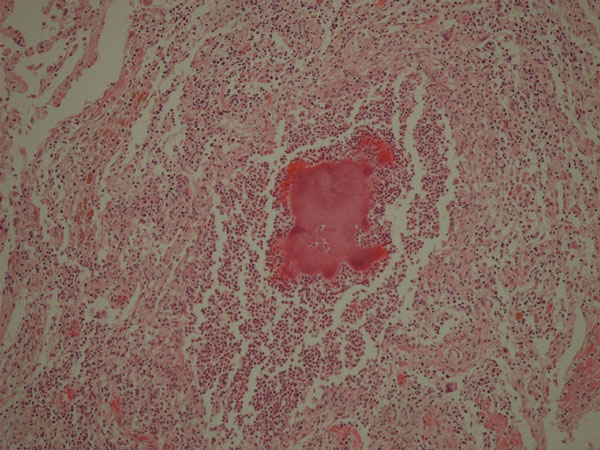
**Intraalveolar active inflammatory response associated with actinomycotic granules**. Hematoxylin and Eosin, 100X.

The patient was a 63-year-old Caucasian woman who presented with a history of recent weight loss, diarrhoea, leg swelling, shortness of breath and depression. GP treated her with Fluoxetine (20 mg), Flucloxacillin (250 mg), Amoxicillin (500 mg), Prednisolone (15 mg), Combivent and Furosemide (40 mg). The patient was a smoker. At the age of 69 her father suffered a brain stroke and from the age of 18 her son was treated for asthma. There was no history of intrauterine contraceptive device use. She was admitted to hospital three days later as hypotensive with abdominal tenderness and vaginal discharge. Laboratory investigations showed microcytic anaemia, low albumin and high white cell count (Table 1). The blood culture turned MRSA negative. A vaginal swab and endometrial pipelle biopsy were also done. CT scans revealed small bowel dilatation, sigmoid diverticula, ascites and pelvic fluid. Treatment included blood transfusion, Dobutamine, iron sulphate, Metronidazole (500 mg), Benzylpenicillin (1.2 g), Gentamycin (700 mg) and Ciprofloxacin (500 mg). The patient died four weeks after admission. A coroner's post mortem examination was requested. Autopsy tissue samples were processed and prepared by standard histological techniques, including Brown-Hopps and PAS on selected samples. The vaginal swab was negative for actinomyces. The pipelle showed a superficially inflamed endometrium with a few actinomycotic granules (Figure 1). Post mortem examination revealed widespread sigmoid diverticular disease and bowel perforation. Pelvic organs were embedded in greenish-yellow pus. The lungs had granular cut surfaces with grey-yellow areas of consolidation. Microscopic examinations of the sigmoid confirmed deep-wall diverticuli showing a frequent intense inflammation, effacement of the mucosal lining, foreign body reaction and actinomycotic granules in inflammatory luminal debris (Figures 2,3). Sections of pelvic structures showed profuse fibrino-purulent exudate containing also numerous actinomycotic granules and remnants of food (Figure 4), affecting only the outer surface of the uterus, often close to the medium sized blood vessels. Lung sections featured bronchopneumonia, focally associated with actinomycotic granules (Figure 5). An extensive tissue search failed to confirm actinomycotic granules in any other organ. Post mortem sampling for microbiological culture of Actinomyces was unsuccessful.

**Table 1:** Summary of laboratory test results

Test	Result	Reference range
White cell count	15.9 10*9/L	4 - 11
Red Cell Count	3.05 10*12/L	3.8 - 5.8
Haemoglobin	8.5 g/dL	11.0 - 16.5
Haematocrit	0.252 1/1	0.37 - 0.51
RBC Distribution Width	16.6%	11.8 - 14.8
Neutrophils (Automated)	14.5 10*9/L	2.0 - 7.5
Lymphocytes (Automated)	1.15 10*9/L	1.5 - 4.0
Albumin	14 g/L	34 - 50

## Discussion

Abdominal actinomycosis has been occasionally reported in the medical literature as a complication of perforated diverticular disease but it is possible that the true incidence is underestimated [1]. Actinomyces require devitalized tissue and a break in the integrity of the mucous membranes to invade, and diverticular disease offers all the conditions for development of infection. Clinical course and histomorphological findings in this case suggest the perforating sigmoid diverticular actinomycosis as an origin of the systemic infection. However, the coexisting endometrial and diverticular actinomycotic disease could not be completely ruled out. Awareness of the possibility of actinomycosis in diverticular disease is important as hematogenous spread can occur at any stage and management of the disease is complex with an uncertain outcome.

## Consent

As this is a coroner's post mortem case, the consent for publishing this case has been received from HM Coroner. We believe this case report contains a worthwhile clinical lesson which could not be as effectively made in any other way. We expect the patient's next-of-kin not to object to the publication since every effort has been made so that the patient remains anonymous.

## Competing interests

The authors declare that they have no competing interests.

## Authors' contributions

AV drafted the manuscript, performed post mortem and microscopic examination of histological samples. KL analysed and presented a summary of patient's notes. All authors read and approved the final manuscript.
